# The Extracellular Domain of the β_2_ Integrin β Subunit (CD18) Is Sufficient for Escherichia coli Hemolysin and Aggregatibacter actinomycetemcomitans Leukotoxin Cytotoxic Activity

**DOI:** 10.1128/mBio.01459-19

**Published:** 2019-07-09

**Authors:** Laura C. Ristow, Vy Tran, Kevin J. Schwartz, Lillie Pankratz, Andrew Mehle, John-Demian Sauer, Rodney A. Welch

**Affiliations:** a Department of Medical Microbiology and Immunology, University of Wisconsin—Madison, Madison, Wisconsin, USA; New York University School of Medicine; University of Oklahoma Health Sciences Center; Children's Hospital of Philadelphia

**Keywords:** cytotoxicity, hemolysin, integrins, leukotoxin, RTX toxin

## Abstract

The Escherichia coli hemolysin (HlyA) is a pore-forming exotoxin associated with severe complications of human urinary tract infections. HlyA is the prototype of the repeats-in-toxin (RTX) family, which includes LtxA from Aggregatibacter actinomycetemcomitans, a periodontal pathogen. The existence and requirement for a host cell receptor for these toxins are controversial. We performed an unbiased forward genetic selection in a mutant library of human monocytic cells, U-937, for host factors involved in HlyA cytotoxicity. The top candidate was the β_2_ integrin β subunit. Δβ_2_ cell lines are approximately 100-fold more resistant than wild-type U-937 cells to HlyA, but remain sensitive to HlyA at high concentrations. Similarly, Δβ_2_ cells are more resistant than wild-type U-937 cells to LtxA, as Δβ_2_ cells remain LtxA resistant even at >1,000-fold-higher concentrations of the toxin. Loss of any single β_2_ integrin α subunit, or even all four α subunits together, does not confer resistance to HlyA. HlyA and LtxA bind to the β_2_ subunit, but not to α_L_, α_M_, or α_X_ in far-Western blots. Genetic complementation of Δβ_2_ cells with either β_2_ or β_2_ with a cytoplasmic tail deletion restores HlyA and LtxA sensitivity, suggesting that β_2_ integrin signaling is not required for cytotoxicity. Finally, β_2_ mutations do not alter sensitivity to unrelated pore-forming toxins, as wild-type or Δβ_2_ cells are equally sensitive to Staphylococcus aureus α-toxin and Proteus mirabilis HpmA. Our studies show two RTX toxins use the β_2_ integrin β subunit alone to facilitate cytotoxicity, but downstream integrin signaling is dispensable.

## Abstract (precis)

Urinary tract infections are one of the most common bacterial infections worldwide. Uropathogenic Escherichia coli strains are responsible for more than 80% of community-acquired urinary tract infections. Although we have known for nearly a century that severe infections stemming from urinary tract infections, including kidney or bloodstream infections are associated with expression of a toxin, hemolysin, from uropathogenic Escherichia coli, how hemolysin functions to enhance virulence is unknown. Our research defines the interaction of hemolysin with the β_2_ integrin, a human white cell adhesion molecule, as a potential therapeutic target during urinary tract infections. The E. coli hemolysin is the prototype for a toxin family (RTX family) produced by a wide array of human and animal pathogens. Our work extends to the identification and characterization of the receptor for an additional member of the RTX family, suggesting that this interaction may be broadly conserved throughout the RTX toxin family.

## INTRODUCTION

Escherichia coli strains include members of our commensal microbiota, as well as pathogens of the intestinal tract, meninges, or urinary tract. Uropathogenic E. coli (UPEC) strains are the causative agent of >80% of community-acquired urinary tract infections (UTIs), which average 8 to 9 million cases annually in the United States, and >50% of hospital-acquired UTIs, which average nearly 1 million cases annually (1, 2). Nearly a century ago, epidemiological studies revealed that UPEC strains were more likely than commensal E. coli to express a hemolytic factor, now known as hemolysin (HlyA) (3). The presence of the *hlyA* gene in the genome of clinical isolates correlates with severity of infection as *hlyA* is carried in 31 to 48% of E. coli strains recovered from uncomplicated UTIs, but in pyelonephritis or urosepsis isolates, 50 to 78% of strains contain the gene (4–6). A precise role for HlyA in progression of these infections is undefined in experimental models. Significant differences in exfoliation and hemorrhage in the murine bladder at early time points are observed in mice intraurethrally inoculated with HlyA-expressing compared to HlyA-deficient UPEC, but no significant differences were observed in colonization or dissemination to the kidneys (7). The importance of HlyA as a virulence factor is clear, as it enhances lethal sepsis following intravenous inoculation, but an animal model of progression to urosepsis from a urinary tract infection does not exist (8).

A precise mechanism for the cytotoxic activity of HlyA has remained controversial despite decades of research. *In vitro*, HlyA is cytotoxic to a wide range of hosts and cell types at high concentrations, fortifying the argument that the pore-forming protein has nonspecific cytotoxic activity (3, 9, 10). At sublytic concentrations, stable insertion of HlyA in the membrane induces changes in the host cell, including inactivation of an important host cell regulator, Akt, induction of the serine protease, mesotrypsin, and activation of caspases—all activities through which the toxin may contribute to UPEC virulence independent of direct lytic destruction of tissue (11, 12). In 1997, Lally et al., using a panel of antibodies against HL-60 surface proteins, identified an HlyA cytolysis-blocking antibody that bound the α_L_β_2_ integrin heterodimer (the CD11a/CD18 [encoded by *ITGAL/ITGB2*] subunits of LFA-1 [i.e., lymphocyte function-associated antigen-1]) (13). Ectopic expression of the heterodimeric integrin on K562 cells that do not natively express β_2_ integrins conferred increased sensitivity to HlyA cytotoxic activity (13). Additionally, Morova et al. determined that the glycosylation state of α_L_β_2_-expressing cells is important for HlyA cytotoxic activity (14). In contrast, Valeva et al. contend that the expression of α_L_β_2_ enhances sensitivity of cells to all pore-forming toxins, suggesting that HlyA activity is not receptor dependent (15). Most recently, Munksgaard et al. demonstrated that K562 cells transfected with α_L_β_2_ are no more sensitive to HlyA than the parental cell line (16). Although the interaction of HlyA with β_2_ integrins has been described in several reports, the significance and reproducibility of this across the field have remained debatable and the assessment of HlyA interaction with each member of the β_2_ integrin family incomplete.

HlyA is the prototypical member of the RTX (repeats-in-toxin) family, a large family of conserved proteins found across Gram-negative bacteria (17). Like HlyA, several other RTX toxin family members found in human-pathogenic bacteria have reported β_2_ integrin family specificity (13). LtxA is expressed by the human pathogen Aggregatibacter actinomycetemcomitans, which can cause a variety of infections. The importance of LtxA as a virulence factor is most apparent in A. actinomycetemcomitans associated with an aggressive form of periodontitis in young adults, as the toxin is produced at 10- to 20-fold-higher levels than in other infectious isolates (18, 19). Similar to historical HlyA reports, literature describing the specificity of LtxA for the α_L_β_2_ integrin heterodimer is controversial, as Dileepan et al. define the specificity of LtxA for the β_2_ subunit alone, whereas in multiple reports, the specificity of LtxA for the complete α_L_β_2_ integrin heterodimer or the α_L_ subunit, narrowed to specific β-sheets of the α_L_ subunit, is described (13, 20–22). LtxA activity can be inhibited with peptides generated based on the defined interacting domain of LtxA with α_L_-subunit β-sheets (22). Additionally, Nygren et al. described the interaction of LtxA with the cytoplasmic domains of the α_L_β_2_ integrin heterodimer, hypothesized to follow internalization of LFA-1/LtxA after the initial extracellular interaction (22, 23). Similar to studies with HlyA, LtxA has been described to interact with β_2_ integrins in nonnative β_2_-expressing cells in some studies, but a thorough examination of the repertoire of β_2_ integrin-LtxA interactions in the context of native β_2_ integrin expression has not been assessed.

In this work, we performed an unbiased genome-wide positive selection in the U-937 human monocytic cell line to identify host factors that contribute to the cytotoxic activity of HlyA. The top hit from our selection was the β subunit of the β_2_ integrin family. We have characterized that for HlyA and LtxA, the presence of the β_2_ integrin β subunit alone is sufficient to enhance cytotoxic activity of the toxins. Additionally, signaling downstream of the β_2_ subunit is not necessary for HlyA- or LtxA-mediated cytotoxicity, as a complemented strain expressing a cytoplasmic tail-deficient β subunit is equally sensitive to HlyA and LtxA cytotoxic activities. Our study provides a thorough examination of the importance of β_2_ integrins in the context of HlyA- or LtxA-mediated cytotoxicity and may provide therapeutic targets for disrupting toxin interactions with the host for both pathogenic bacteria.

## RESULTS

### GeCKO library selection identified host factors that contribute to HlyA cytotoxic activity.

**FIG 1:**
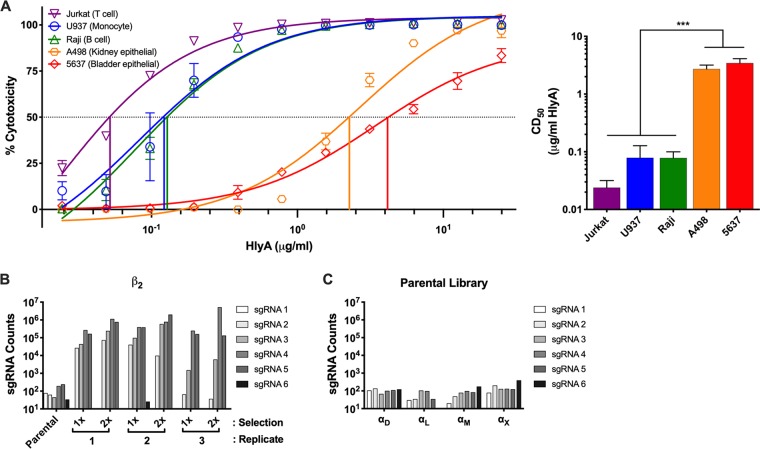
Specific host factors contribute to hemolysin cytotoxic activity. (A) (Left) PEG-precipitated HlyA was incubated at various concentrations with cell lines at 1 × 10^6^ cells/ml for 1 h. Cells were washed, and cell viability was measured by XTT assay. The percentage of cytotoxicity was normalized to Triton X-100-treated cells at 100% and RPMI-only-treated cells at 0%. (Right) The CD_50_ was calculated in GraphPad Prism, and bars represent the average and standard error of the mean (SEM) from 3 biological replicates. One-way analysis of variance (ANOVA) with Bonferroni’s multiple-comparison test was performed in GraphPad Prism. ***, *P* < 0.001. (B) Six unique sgRNAs targeting β_2_ were present in the GeCKO library. Individual sgRNA counts were normalized to total read counts for each selection and replicate. Counts from the parental library and three replicates are represented after the first and second selections with HlyA. (C) Six unique sgRNAs targeting each of the β_2_ family α subunits were present in the GeCKO library. Individual sgRNA counts were normalized to total read counts for the parental library. Bars represent the counts from a single parental library sgRNA count. Two individual aliquots of the parental library were sequenced and are internally consistent.

Multiple human cell lines historically used in UPEC research were examined for susceptibility to HlyA, including human bladder epithelial cells (5637), human kidney epithelial cells (A498), human T lymphocytes (Jurkat), human B lymphocytes (Raji), and human monocytes (U-937). The cytotoxic activity of HlyA was characterized with HlyA at a range of concentrations, and the state of cellular redox potential as a proxy for cell viability was monitored by XTT [2,3-bis-(2-methoxy-4-nitro-5-sulfophenyl)-2H-tetrazolium-5-carboxanilide salt] assay (Fig. 1A). As previously described, HlyA was cytotoxic at high concentrations across all cell types examined, but interestingly, differences in the concentration at which 50% of cells are killed (cytotoxic dose 50 [CD_50_]) ranged up to 100-fold, suggesting the existence of factors that contribute to cell line specificity (3, 9, 10).

To identify host-specific factors required for the cytotoxic activity of HlyA, we focused on one of the most HlyA-sensitive lines examined, U-937 cells (Fig. 1A). We generated a Genome-scale CRISPR Knock-Out (GeCKO) library using the GeCKO v2 library developed in the Zhang lab (24). This library contains over 120,000 single guide RNAs (sgRNAs), with 6 sgRNAs targeting each of 19,050 genes and 4 sgRNAs targeting each of 1,864 microRNAs (miRNAs) (24). We performed two serial selections of 6 × 10^7^ cells (500× representation of each sgRNA) of the U-937 mutant library with a concentration of HlyA at which nearly 100% of parental U-937 cells were killed, in biological triplicate (see Fig. S1 in the supplemental material). Genomic DNA was isolated, and deep sequencing of amplified and barcoded sgRNAs from each replicate was performed to identify enriched sgRNAs in both the single- and double-treated populations compared to the untreated parental library. Enriched sgRNAs were ranked using the Model-based Analysis of Genome-scale CRISPR-Cas9 Knockout (MAGeCK) tool, which was exclusively designed to analyze results from GeCKO library selections (25). Four of the six sgRNAs targeting the integrin subunit β_2_ gene (*ITGB2*) were positively selected in all HlyA-treated libraries, comprising up to 84% of the sequence reads from one of the populations and generating the most robust hit by several orders of magnitude in our selection (Fig. 1B; see Fig. S2 in the supplemental material). The β_2_ integrin family includes the surface-expressed heterodimeric proteins α_D_β_2_ (CD11d/CD18 [encoded by *ITGAD/ITGB2*]), α_L_β_2_ (CD11a/CD18 [encoded by *ITGAL/ITGB2*], subunits of LFA-1 [lymphocyte function associated antigen-1]), α_M_β_2_ (CD11b/CD18 [encoded by *ITGAM/ITGB2*], subunits of Mac-1 [macrophage-1 antigen]), and α_X_β_2_ (CD11c/CD18 [encoded by *ITGAX/ITGB2*], p150/95)_,_ all of which are exclusively expressed on leukocytes (26). Despite comparable representation in the parental libraries of sgRNAs targeting each of the four α subunits relative to the β_2_ subunit (Fig. 1B and 1C), none were enriched across three biological replicates (see Fig. S3 in the supplemental material). Taken together, the selection and sequencing results suggest that at least one isoform of the β_2_ integrin family mediates increased sensitivity of U-937 cells to HlyA activity, but that no single αβ pair is necessary.

10.1128/mBio.01459-19.1 FIG S1 Library selection. (A) Work flow for library selection. The parental library was sequenced in duplicate, and HlyA selections were performed in biological triplicate. (B) Culture supernatant from E. coli K-12 expressing HlyA was incubated at various concentrations with U-937 cells at 1 × 10^6^ cells/ml for 1 h. Cells were washed, and cell viability was measured by XTT assay. The percentage of cytotoxicity was normalized to Triton X-100-treated cells at 100% and LB-treated cells at 0%. Data points represent the average and SEM from technical duplicates. The asterisk indicates the concentration used for library selection. Download FIG S1, TIF file, 0.4 MB. Copyright © 2019 Ristow et al. 2019 Ristow et al. This is an open-access article distributed under the terms of the Creative Commons Attribution 4.0 International license.

10.1128/mBio.01459-19.2 FIG S2 The β_2_ (*ITGB2*) gene is the most robust positively selected gene for resistance to HlyA cytotoxic activity. sgRNA counts were analyzed in MAGeCK from three biological replicates of library selection. The robust ranking analysis (RRA) score is reported with SEM represented, and 1, 5, and 10% false-discovery rates (FDR) are indicated by dotted lines. Download FIG S2, TIF file, 0.3 MB. Copyright © 2019 Ristow et al. 2019 Ristow et al. This is an open-access article distributed under the terms of the Creative Commons Attribution 4.0 International license.

10.1128/mBio.01459-19.3 FIG S3 β_2_ integrin family α subunits are not necessary for HlyA cytotoxic activity. Six unique sgRNAs targeting each α subunit of the β_2_ integrin family were present in the GeCKO library. Individual sgRNA counts for α_D_, α_L_, α_M_, and α_X_ were normalized to total read counts for each selection and replicate. Counts from the parental library (data also represented in Fig. 1C), and three replicates are represented after the first and second selections with HlyA. Download FIG S3, TIF file, 0.3 MB. Copyright © 2019 Ristow et al. 2019 Ristow et al. This is an open-access article distributed under the terms of the Creative Commons Attribution 4.0 International license.

### Enhanced HlyA cytotoxic activity in the presence of the β_2_ integrin family is redundant among different α subunits.

**FIG 2:**
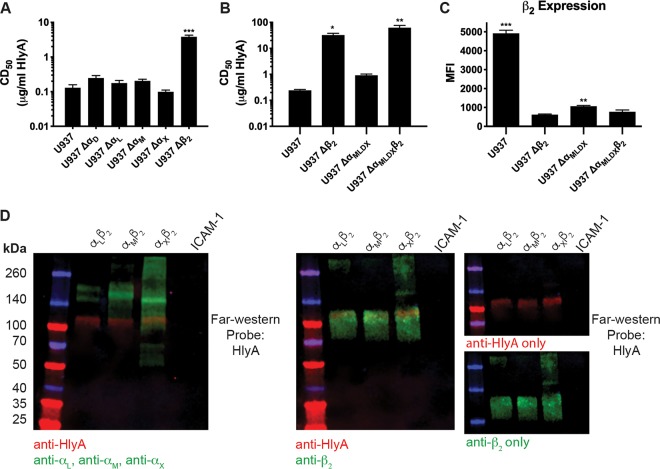
β_2_ integrin β subunit is necessary for U-937 cell sensitivity to HlyA cytotoxic activity. (A) PEG-precipitated HlyA was incubated at various concentrations with the U-937 wild-type and individual-integrin-subunit-knockout cell lines at 2 × 10^6^ cells/ml for 1 h. Cells were washed, and cell viability was measured by XTT assay. The percentage of cytotoxicity was normalized to Triton X-100-treated cells at 100% and RPMI-only-treated cells at 0%. The CD_50_ was calculated in GraphPad Prism, and bars represent the average and SEM from 3 biological replicates. One-way ANOVA with Bonferroni’s multiple-comparison test was performed in GraphPad Prism, with significance of each cell type compared to U-937 cells represented. (B) As in panel A, a standard XTT cytotoxicity assay was performed with multiple-integrin-subunit-knockout cell lines as indicated. Results were normalized and statistics determined as described above. (C) β_2_ expression was assessed by flow cytometry on intact cells from the cell lines indicated. Bars represent the average mean fluorescent intensity (MFI) and SEM from 3 biological replicates with at least 50,000 events recorded per replicate. One-way ANOVA with Bonferroni’s multiple-comparison test was performed in GraphPad Prism, with the significance of each cell type compared to U-937 Δβ_2_ cells represented. *, *P* < 0.05; **, *P* < 0.01; ***, *P* < 0.001. (D) Recombinant integrin pairs and human ICAM-1 were separated by a 4 to 20% gradient SDS-PAGE gel, transferred to nitrocellulose, and probed with HlyA at 1 μg/ml. (Left) Bound HlyA was detected with polyclonal anti-HlyA, and integrin α subunits were detected with monoclonal antibodies. (Center) Bound HlyA was detected with a pool of monoclonal anti-HlyA antibodies, and the integrin β_2_ subunit was detected with a polyclonal antibody. (Right) Single-channel images of the center blot. Multiplexed near-infrared fluorescence was used to detect multiple proteins on the same blot using a Licor Odyssey imager. Blots are representative of three biological replicates.

We generated clonal targeted CRISPR/Cas9-mediated knockout U-937 cells deficient in each of the four individual α subunits and the β_2_ subunit, using 3 unique sgRNA sequences per subunit from the GeCKO v2 library. Potential clones were screened for disruption of the target gene by IDAA (indel detection by amplicon analysis) and loss of target gene expression by flow cytometry and confirmed by sequencing (see Table S1 in the supplemental material) (27). The HlyA CD_50_ was determined for each clonal knockout cell line as in Fig. 1. No difference in sensitivity to HlyA was observed in any Δα cell line, which confirmed selection results that no single α subunit is necessary for HlyA activity (Fig. 2A). In contrast, a clonal Δβ_2_ cell line demonstrated nearly a 100-fold increase in CD_50_ of HlyA (Fig. 2A), which correlated with our selection results and was consistent with the difference between HlyA cytotoxicity in U-937 cells compared to human bladder or kidney epithelial cells (natively β_2_^−/−^) (Fig. 1A).

10.1128/mBio.01459-19.5 TABLE S1 Table of mutations in knockout cell lines. Download Table S1, PDF file, 0.03 MB. Copyright © 2019 Ristow et al. 2019 Ristow et al. This is an open-access article distributed under the terms of the Creative Commons Attribution 4.0 International license.

To assess the sufficiency of each αβ pair in mediating sensitivity to HlyA, we set out to generate cell lines deficient in all combinations of two, three, or all four α subunits to assess the effect on the HlyA CD_50_. Confirmed single-α knockouts that constitutively express the Cas9 nuclease were electroporated with sgRNA to target additional α subunits. Multi-α knockouts were generated in a stepwise fashion, with single-cell clones obtained and knockouts confirmed for each α subunit in turn. Although we generated multiple combinations of double (Δα_DL_, Δα_DX_, Δα_LM_, and Δα_LX_) and triple (Δα_DLM_, Δα_DMX_) α subunit knockouts and hypothesized that the triple α subunit knockouts would be the most informative regarding the specificity or redundancy between the β_2_ family integrins for HlyA activity, a quadruple α subunit knockout revealed the most unexpected result. Integrins are characterized as requisite heterodimers for functional surface expression (28, 29). Therefore, we hypothesized that a quadruple α subunit knockout (Δα_DLMX_) would phenocopy a β_2_ knockout both in a complete loss of surface expression of β_2_ and in increased resistance to HlyA. Interestingly, a quadruple α knockout cell line only had a minor increase in resistance to HlyA compared to wild-type U-937 cells (Fig. 2B) and retained a small population of β_2_ on the cell surface, observed by flow cytometry on intact cells (Fig. 2C). In the Δα_DLMX_ background, we confirmed that the observed phenotype was due to the presence of β_2_ and not unintentional mutations acquired during the mutagenesis strategy by additionally knocking out the β_2_ subunit (Δα_DLMX_β_2_). The Δα_DLMX_β_2_ cell line phenocopies a single β_2_ subunit knockout both in resistance to HlyA cytotoxic activity (Fig. 2B) and loss of β_2_ expression (Fig. 2C), confirming that the presence of β_2_ confers sensitivity to HlyA cytotoxic activity.

The sensitivity of the U-937 Δα_DLMX_ cells together with the resistance of the Δα_DLMX_β_2_ cells suggested that HlyA interaction with β_2_, in the absence of any known α subunit binding partners, was sufficient to confer sensitivity. To test this hypothesis, we performed a far-Western blot with HlyA against various β_2_ integrin heterodimers. Far-Western blots with recombinant α_L_β_2_, α_M_β_2_, and α_X_β_2_ protein demonstrate that HlyA interacts directly with the β_2_ subunit, but not any of the α subunits (Fig. 2D). The resistance of β_2_ integrin-deficient mutant cells combined with far-Western blotting suggests that HlyA interaction with the β_2_ integrin β subunit is sufficient for cytotoxicity in U-937 cells.

### β_2_ subunit enhancement of HlyA cytotoxic activity is not dependent on downstream signaling.

**FIG 3:**
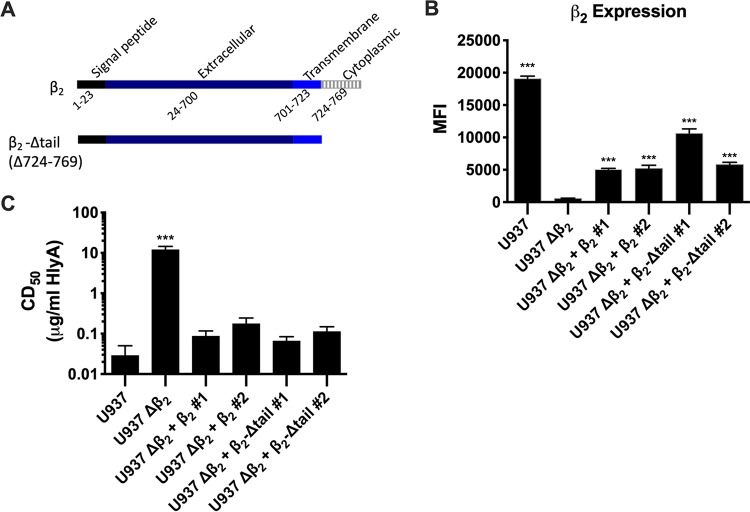
Signaling downstream of β_2_ is not required for sensitivity of U-937 cells to HlyA cytotoxic activity. (A) Schematic diagram of *ITGB2* full-length protein and cytoplasmic-tail deletion. (B) β_2_ expression was assessed by flow cytometry on intact cells from the wild-type and Δβ_2_ lines and two clones each of the complemented Δβ_2_ plus wild-type β_2_ or Δβ_2_ plus Δtail-β_2_ cell lines. Bars represent the average median fluorescence intensity (MFI) and SEM from 3 biological replicates with at least 50,000 events recorded per replicate. One-way ANOVA with Bonferroni’s multiple-comparison test was performed in GraphPad Prism, with significance of each cell type compared to U-937 Δβ_2_ cells represented. (C) PEG-precipitated HlyA was incubated at various concentrations with cell lines described in panel B at 2 × 10^6^ cells/ml for 1 h. Cells were washed, and cell viability was measured by XTT assay. The percentage of cytotoxicity was normalized to Triton X-100-treated cells at 100% and RPMI-only-treated cells at 0%. The CD_50_ was calculated in GraphPad Prism, and bars represent the average and SEM from 3 biological replicates. One-way ANOVA with Bonferroni’s multiple-comparison test was performed in GraphPad Prism, with significance of each cell type compared to U-937 cells represented. ***, *P* < 0.001.

To determine if β_2_ integrin signaling is required for HlyA-induced cytotoxicity, we generated U-937 Δβ_2_ complemented strains with either wild-type β_2_ or a β_2_ subunit lacking the cytoplasmic tail (Fig. 3A). Although we were unable to achieve wild-type levels of β_2_ expression on the surface with either wild-type or the cytoplasmic tail deletion (Fig. 3B), both complements restore sensitivity to HlyA activity, suggesting that the cytotoxic activity of HlyA requires little β_2_ on the surface of cells and does not require signaling downstream of β_2_ integrins (Fig. 3C).

### The β_2_ integrin β subunit specifically enhances cytotoxic activity of a related RTX toxin.

**FIG 4:**
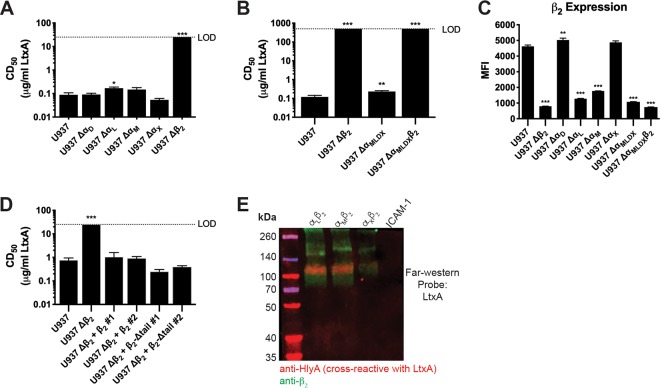
β_2_ integrins specifically enhance the activity of LtxA. (A, B, and D) PEG-precipitated LtxA was incubated at various concentrations with U-937 wild-type, individual- or multiple-subunit-knockout cell lines, or complemented Δβ_2_ cells as indicated for each panel at 2 × 10^6^ cells/ml for 3 h. Following toxin incubations, cells were washed, and cell viability was measured by XTT assay. The percentage of cytotoxicity was normalized to Triton X-100-treated cells at 100% and RPMI-only-treated cells at 0%. The CD_50_ was calculated in GraphPad Prism, and bars represent the average and SEM from 3 biological replicates. The dashed line indicates the limit of detection. One-way ANOVA with Bonferroni’s multiple-comparison test was performed in GraphPad Prism, with significance of each cell type compared to U-937 cells represented. *, *P* < 0.05; **, *P* < 0.01; ***, *P* < 0.001. (C) β_2_ expression was assessed by flow cytometry on intact cells from the cell lines indicated. Bars represent the average MFI and SEM from 3 biological replicates with at least 50,000 events recorded per replicate. One-way ANOVA with Bonferroni’s multiple-comparison test was performed in GraphPad Prism, with significance of each cell type compared to U-937 cells represented. *, *P* < 0.05; **, *P* < 0.01; ***, *P* < 0.001. (E) Recombinant integrin pairs and human ICAM-1 were separated by a 4 to 20% gradient SDS-PAGE gel, transferred to nitrocellulose, and probed with LtxA at 1 μg/ml. Bound LtxA was detected with cross-reactive monoclonal anti-HlyA antibodies. The integrin β_2_ subunit was detected with a polyclonal antibody. Multiplexed near-infrared fluorescence was used to detect multiple proteins on the same blot using a Licor Odyssey imager. Blot is representative of three biological replicates.

As the prototypic member of the RTX toxin family, HlyA’s structure and function are shared by related toxins present in many different human and animal pathogens. To determine the requirement of β_2_ integrins for an additional RTX toxin family member, we utilized the mutants described above to assess sensitivity to LtxA from Aggregatibacter actinomycetemcomitans. The indicated cell lines were challenged with LtxA for 3 h before determination of the CD_50_. Unlike HlyA, LtxA activity is redundant across three of the four α subunits compared to wild-type U-937 cells (Fig. 4A), but is significantly reduced in the absence of α_L_ or of all four α subunits, Δα_DLMX_ (Fig. 4A and B). The increased resistance of these cell lines is inversely correlated with the expression of β_2_ on their cell surface (Fig. 4C and 2C). A dramatic increase in resistance to LtxA is observed in the absence of the β subunit of the β_2_ integrin family, as no activity was observed on Δβ_2_ or Δα_DLMX_β_2_ U-937 cells despite being treated with >1,000-fold concentrations higher than the CD_50_ of LtxA on wild-type U-937 cells (Fig. 4B; see Fig. S4 in the supplemental material). The dependence of LtxA on the presence and abundance of the β subunit on the surface of cells for cytotoxic activity is supported by a far-Western blot analysis of LtxA interaction with recombinant integrins, in which LtxA phenotypically copied HlyA with interaction solely with the β subunit (Fig. 4D). Complementation of Δβ_2_ U-937 cells with the wild type or a cytoplasmic tail deletion of the β_2_ subunit restores sensitivity to LtxA cytotoxic activity, indicating that like HlyA, LtxA does not require signaling downstream of β_2_ integrins (Fig. 4E).

10.1128/mBio.01459-19.4 FIG S4 LtxA is not active against U-937 cells lacking β_2_. PEG-precipitated LtxA was incubated at various concentrations with U-937 wild-type and multiple-integrin-subunit-knockout cell lines at 2 × 10^6^ cells/ml for 3 h. Cells were washed, and cell viability was measured by XTT assay. The percentage of cytotoxicity was normalized to Triton X-100-treated cells at 100% and RPMI-only-treated cells at 0%. Data points represent the average and SEM from technical duplicates. Data are representative of experiments performed in biological triplicate. Download FIG S4, TIF file, 0.3 MB. Copyright © 2019 Ristow et al. 2019 Ristow et al. This is an open-access article distributed under the terms of the Creative Commons Attribution 4.0 International license.

### The enhancement of cytotoxic activity in the presence of β_2_ integrins does not extend to all pore-forming toxins.

**FIG 5:**
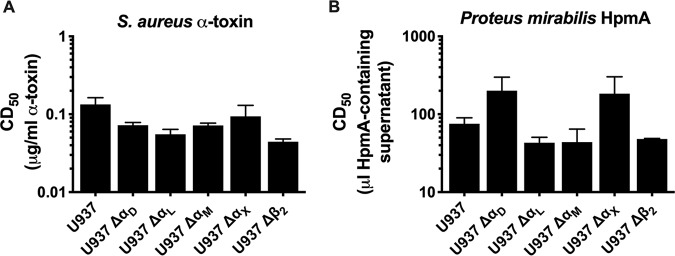
β_2_ integrins do not enhance the cytotoxic activity of all pore-forming toxins. (A) Purified Staphylococcus aureus α-toxin was incubated at various concentrations with the U-937 wild-type or individual-subunit-knockout cell lines as indicated for each panel at 2 × 10^6^ cells/ml for 24 h. (B) Supernatant from recombinant E. coli K-12 expressing Proteus mirabilis HpmA was incubated at various concentrations with the U-937 wild-type or individual-subunit-knockout cell lines at 2 × 10^6^ cells/ml for 1 h. Following all toxin incubations, cells were washed, and cell viability was measured by XTT assay. The percentage of cytotoxicity was normalized to Triton X-100-treated cells at 100% and RPMI-only-treated cells at 0%. The CD_50_ was calculated in GraphPad Prism, and bars represent the average and SEM from 3 biological replicates. One-way ANOVA with Bonferroni’s multiple-comparison test was performed in GraphPad Prism, with significance of each cell type compared to U-937 cells represented (none found).

Previous work suggested that the expression of β_2_ integrins not only increases sensitivity of cells to HlyA, but also increases it to multiple, unrelated pore-forming toxins (15). Although that result strongly supported a non-β_2_-specific activity of HlyA, the investigators relied on the presence and absence of recombinant expression of α_L_β_2_ in nonnative β_2_-expressing cells. To assess the role of β_2_ integrins in sensitizing native β_2_-expressing cells to diverse pore-forming toxins, the indicated cell lines were treated with Staphylococcus aureus α-toxin for 24 h before determination of the CD_50_. Knockout of any α subunit or β_2_ did not significantly affect the sensitivity of cells to α-toxin (Fig. 5A). Similarly, the indicated cell lines were treated with culture supernatant containing the unrelated pore-forming toxin HpmA from Proteus mirabilis for 1 h before determination of the CD_50_. All cell lines were equally susceptible to the pore-forming cytotoxic activity of HpmA, and expression of β_2_ integrins did not significantly alter the CD_50_ (Fig. 5B). These results suggest that the presence of β_2_ integrins does not nonspecifically enhance the cytotoxic activity of pore-forming toxins.

## DISCUSSION

The UPEC HlyA has long been appreciated as an important virulence factor in complicated UTIs that lead to pyelonephritis or urosepsis. Despite the efforts of many different laboratories, the role that this toxin plays in uropathogenesis remains poorly understood. We began this study with side-by-side comparisons of the sensitivity of different cultured human cell types to HlyA. At high concentrations, HlyA is ubiquitously cytotoxic. By examining the cytotoxic effects of HlyA over a range of concentrations, we observed significant differences in the activity of the toxin across a variety of human cell lines, with leukocytes (monocyte-like and lymphocyte-like cells) 100-fold more sensitive to the toxin than either bladder or kidney epithelial cells. This observation provided the rationale to perform a forward genetic screen using CRISPR/Cas9 technology to identify host cell factors involved in HlyA-mediated cytotoxic activity using the human monocyte-like cell line U-937. Despite extensive mutagenic coverage of the entire genome, our selection and enrichment yielded multiple mutant alleles of a single gene, *ITGB2*, which encodes the β subunit of the β_2_ integrin heterodimer.

Although the role of α_L_β_2_ as a specific receptor for HlyA is controversial, several groups demonstrate that the presence of this β_2_ receptor on cells increases sensitivity to HlyA, so we predicted we would observe mutants in genes encoding both α_L_ and β_2_ subunits (13, 15). While we did recover the β subunit of the α_L_β_2_ integrin heterodimer, the absence of α_L_ was unexpected, as integrins canonically function as obligate heterodimers and require an α subunit and β subunit for proper processing and expression on the surface of cells (28, 29). We confirmed that the U-937 mutant library contained multiple guide RNAs targeting the *ITGAL* gene that encodes the α_L_ subunit, but they did not appear in our selected population. Previous work with α_L_β_2_ and several RTX toxins, including HlyA and LtxA, was performed by ectopic expression of α and β_2_ integrin subunit genes in nonleukocyte cells or by the addition of α-encoding cDNA to ethyl methanesulfonate (EMS)-mutagenized Jurkat cells that lacked β_2_ surface expression (13, 15, 30). Perhaps contributing to the controversy of the role of specific β_2_ integrins in mediating HlyA cytotoxicity, no unbiased assessment of the role of all β_2_ integrins in HlyA-mediated toxicity has been performed. Our selection results indicated that the α subunits are redundant for HlyA cytotoxic activity in that no single αβ pair was necessary for HlyA cytotoxic activity. We proceeded to generate directed mutations in each of the α-subunit-encoding genes and the β_2_ integrin β-subunit-encoding gene. None of the α subunit-encoding gene mutants were altered in HlyA sensitivity, but we characterized the β subunit mutant with a 100-fold increase in HlyA resistance. Although the increase in resistance of the α_L_ subunit mutant to LtxA was statistically significant, the biological relevance of that 2-fold increase is slight compared to the over 1,000-fold increase in LtxA resistance in the β-subunit mutant and is likely due to the significant decrease in the β subunit on the cell surface. Double, triple, and quadruple α subunit-encoding gene mutants also failed to be significantly altered in their HlyA sensitivity compared to the parental U-937 cells.

Earlier observations demonstrated that in natively expressing β_2_ integrin leukocytes, β_2_ integrins appear to require intracellular heterodimer formation for processing and glycosylation before there is cell surface expression (28, 29). We therefore were surprised that there was detectable β_2_ subunit expression on the quadruple α subunit mutants. Upon further genetic manipulation and biochemical confirmation, we observed direct interaction with the β subunit and that the presence of the β subunit alone is enough to facilitate increased sensitivity to the cytotoxic activity of HlyA and LtxA. This work helps to clarify long-standing controversies in the HlyA and LtxA fields regarding the presence or absence of a receptor, although understanding the mechanism by which the interaction of either of these toxins with the β subunit enhances cytotoxic activity or the specific molecular interactions between the proteins remains to be understood.

Complementation of the *ITGB2* mutants with either full-length *ITGB2* or an *ITGB2* recombinant missing the cytoplasmic tail of the β subunit was sufficient to confer sensitivity to RTX toxins HlyA and LtxA. The cytoplasmic tail is necessary for outside-in signaling via β_2_ integrins when they engage their extracellular ligands (31). Therefore, we conclude that at least these two RTX toxins, HlyA and LtxA, engage and use the extracellular portion of the β_2_ integrin β subunit to facilitate cytotoxicity. There are several different physiological effects that occur when cells are challenged with sublytic concentrations of HlyA; however, our results do not indicate whether signaling via the β subunit is involved in any of these events (12, 32, 33). Further investigation is required to clarify whether pore formation, facilitated by the β subunit interaction or independent of that, is the mechanism at the heart of sublytic host cell events.

Our results demonstrate that although the β_2_ integrin β subunit enhances HlyA cytotoxic activity, HlyA cytotoxic activity is still observed on β_2_-deficient cells at higher doses of toxin. This suggests that there may be a secondary receptor or receptor-independent activity for HlyA. At this point, we do not know if the host cellular events that occur with the HlyA β_2_ integrin β subunit-dependent and -independent activities are similar. It is remarkable that for the related RTX toxin, LtxA cytotoxic activity is inversely linked with the surface expression of the β_2_ integrin β subunit and that LtxA does not show detectable cytotoxic activity on the *ITGB2* mutants at the highest LtxA concentrations that we can acquire. This suggests that LtxA either does not interact with a secondary receptor or lacks the receptor-independent activity. There is one significant phenotypic difference, erythrolysis, which differentiates HlyA and LtxA toxins. HlyA has potent activity toward erythrocytes, whereas LtxA has weak to nonexistent red cell lytic activity (34, 35). Glycophorin, a sialoglycoprotein, has been identified as a receptor for HlyA on erythrocytes, but its expression is limited to that cell type (36). We previously identified an insertion mutant in the HlyA repeat region (HlyA 829::PLQD) that retains 100% of the HlyA erythrolytic activity, but which lacks detectable leukolytic activity against BL3 cells, a bovine lymphoma cell line (37). Thorough genetic and biochemical studies of HlyA and LtxA will be required to identify the receptor-ligand interaction between these toxins and the β_2_ integrin β subunit.

**FIG 6:**
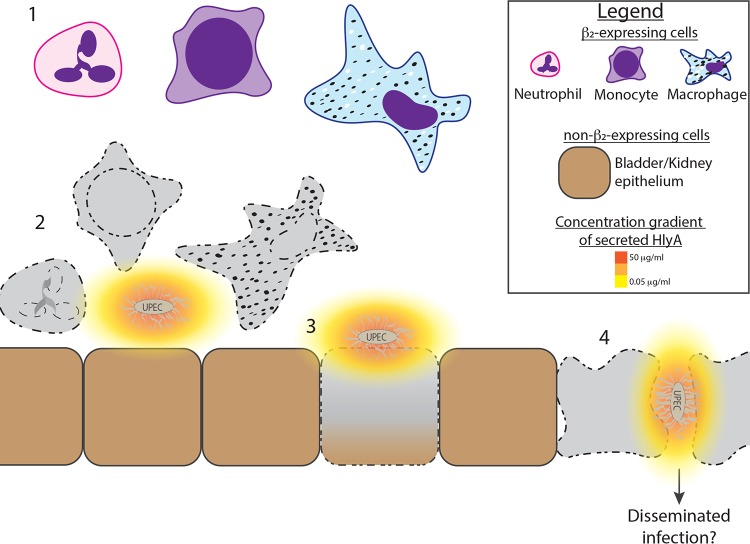
Model for HlyA cytotoxic activity in infected tissue. (Part 1) A variety of leukocytes are recruited to the infected bladder/kidney. (Part 2) Secreted HlyA diffuses away from the UPEC bacteria and forms a gradient. β_2_-expressing leukocytes are sensitive to HlyA at low concentrations, where the toxin exhibits cytotoxic effects on the cells before they can effectively clear the bacteria, whereas non-β_2_-expressing epithelial cells remain viable at low concentrations of the HlyA. (Part 3) Non-β_2_-expressing epithelial cells are susceptible to cytotoxic effects of HlyA at high concentrations, likely at sites of adherent bacteria. (Part 4) Disruption of the epithelial cell barrier may lead to disseminated infection and severe complications from UTIs, like pyelonephritis and urosepsis.

Based on our results, we present a model that may begin to explain how RTX toxins affect the pathogenesis of disease regardless of the specific niche occupied by the RTX toxin-expressing pathogen (Fig. 6). At the heart of the model is the acute sensitivity of leukocytes to these toxins. For HlyA-expressing UPEC strains that colonize the urinary tract, leukocytes may be the first cells to be affected by this extracellular toxin. The need for just the β subunit without any structure provided by an α subunit indicates that all leukocytes are vulnerable whether they are monocytes, neutrophils, lymphocytes, or dendritic cells. Thus, the RTX toxins provide the relevant pathogens a global attack on both the innate and adaptive immune response. In the specific case of the UPEC HlyA exotoxin, its cytotoxic activity against cells lacking cell surface β_2_ integrins suggests to us that when UPEC cells are in close proximity to the epithelial barrier, locally high concentrations of HlyA can disrupt epithelial cells in order to aid deeper tissue invasion and possible systemic spread, supporting the clinical observation of the increased prevalence of HlyA in isolates from pyelonephritis and urosepsis patients. Therapeutically blocking the specific interaction of HlyA with β_2_ integrins may better allow the immune system access to overcome UPEC causing simple cystitis before the infection proceeds to a more severe and disseminated form.

## MATERIALS AND METHODS

### Bacterial strains, culture, and toxin preparations.

Recombinant E. coli strain WAM1824 (described in reference 36), expresses the complete *hlyCABD* operon from a single plasmid (pSF4000), and wild-type HlyA was obtained by culturing this strain to an optical density at 600 nm (OD_600_) of 0.8, pelleting bacterial cells, and filter-sterilizing culture supernatants through a 0.45-μm-pore syringe filter unit (Acrodisc 4508) or concentrating HlyA from culture supernatants by polyethylene glycol (PEG) precipitation as follows. WAM1824 was grown overnight at 37°C on LB agar plates containing 5% sheep red blood cells and 20 μg/ml chloramphenicol. Cells were swabbed from plates into 1 ml 1× PBS and diluted into 400 ml LB containing 20 μg/ml chloramphenicol and 2 mM CaCl_2_ to an OD_600_ of 0.05. Cultures were grown at 37°C with aeration to an OD_600_ of 0.8. Cells were pelleted, and the supernatant was filtered through a 500-ml Nalgene Rapid-Flow aPES filtration unit. Protein was precipitated from supernatants by adding 200 g/liter PEG 3350 and 3% glycerol and stirring gently for 1 h at 4°C. Precipitated protein was pelleted by centrifugation at 9,700 × *g* at 4°C for 10 min. Pellets were resuspended in 0.85% saline, aliquoted in 100-μl single-use aliquots, and stored at −80°C. HlyA concentrations were determined by comparison with a β-galactosidase standard of known concentration on Coomassie-stained polyacrylamide gels.

To obtain wild-type LtxA, Aggregatibacter actinomycetemcomitans strain Penn JP2 (a generous gift from S. Kachlany, originally described in reference 38) was grown for 48 h at 37°C under 4% CO_2_ on AAGM agar (dextrose, sodium bicarbonate, Trypticase soy, yeast extract, and agar) plates. A Δ*lktA* JP2 derivative, generated in our lab by transformation with the suicide plasmid pKD378 (a generous gift from D. Kolodrubetz, described in reference 39), was grown for 48 h at 37°C under 4% CO_2_ on Columbia agar plates containing 5% sheep red blood cells and 100 μg/ml spectinomycin. Cells were swabbed from plates into 1 ml 1× phosphate-buffered saline (PBS) and diluted into 10 ml TSBYE (Trypticase soy broth and yeast extract) containing 1 mM CaCl_2_ to an OD_600_ of 0.05. Cultures were grown at 37°C with very slight aeration overnight to an OD_600_ of 0.4 to 0.6. Overnight cultures were diluted into 400 ml TSBYE containing 1 mM CaCl_2_ and grown overnight at 37°C with shaking at 50 rpm to an OD_600_ of 0.4 to 0.6. Cells were pelleted, and the supernatant was filtered through a 500-ml Nalgene Rapid-Flow aPES filtration unit. Protein was precipitated and LtxA quantified following the protocol outlined above for HlyA. Equivalent culture starting volumes and PEG-precipitated resuspension volumes were used from the Δ*lktA* strain to serve as a control.

To obtain HpmA, recombinant E. coli strain WPM 184 (40, 41) was grown overnight at 37°C on LB agar plates containing 5% sheep red blood cells and 100 μg/ml carbenicillin. Cells were swabbed from plates into 1 ml 1× PBS and diluted into 25 ml LB containing 100 μg/ml carbenicillin to an OD_600_ of 0.05. Cultures were grown at 37°C with aeration to an OD_600_ of 0.8. Cells were pelleted, and the supernatant was filtered through a 0.45-μm syringe filter unit (Acrodisc 4508) before use in cytotoxicity assays.

Staphylococcus aureus α-toxin was obtained from Sigma-Aldrich (catalog no. H9395) and resuspended in distilled water (dH_2_O) according to the manufacturer’s instructions.

### Cell culture.

Human kidney epithelial cells (A498 [42]), human T lymphocytes (Jurkat [43]), human B lymphocytes (Raji [44]), human promonocytic myeloid leukemia cells (U-937 [45]), and human bladder epithelial cells (5637 [46]) were cultured in RPMI 1640 (Gibco) with 10% heat-inactivated fetal bovine serum (Gibco), 1 mM sodium pyruvate (Gibco), 2 mM l-glutamine (Gibco), and 25 mM HEPES (Gibco) at 37°C under 5% CO_2_. Human embryonic kidney epithelial cells, HEK293T (47), were cultured in Dulbecco’s modified Eagle’s medium (DMEM [Corning]) with 10% heat-inactivated fetal bovine serum, 1 mM sodium pyruvate, and 2 mM l-glutamine at 37°C under 5% CO_2_.

### Cytotoxicity assay.

Culture supernatant containing PEG-precipitated RTX toxins and culture supernatant containing HpmA were incubated at various concentrations (2-fold dilutions) with the indicated cell lines at 1 × 10^6^ to 2 × 10^6^ cells/ml for 1 to 3 h at 37°C. S. aureus α-toxin was incubated with the indicated cell lines at 2 × 10^6^ cells/ml for 24 h at 37°C. Cells were washed, and cell metabolism as a proxy for cell viability was measured by a standard XTT assay with colorimetric development of XTT [2,3-bis-(2-methoxy-4-nitro-5-sulfophenyl)-2H-tetrazolium-5-carboxanilide at 0.5mg/ml (Sigma)] with PMS (*N*-methyl dibenzopyrazine methyl sulfate; 3.8 μg/ml [Sigma]) diluted in RPMI medium without phenol red (Sigma) (48). Cells were incubated with XTT solution at 37°C for 1 to 3 h before measuring absorbance at 450 nm. Results were normalized to cells treated with Triton X-100 as 100% death and RPMI alone as 0% death. CD_50_ values were determined, and statistics were performed in GraphPad Prism version 6.0 (GraphPad Software).

### Library construction and selection.

Human CRISPR Knockout Pooled Libraries (lentiCRISPRv2 backbone) no. 1000000048 and 1000000049 (Addgene) were used to generate lentivirus per the Zhang lab protocol (24). U-937 cells were transduced with lentivirus at a multiplicity of infection (MOI) of 0.3. Twenty-four hours posttransduction, cells were selected with 0.5 μg/ml puromycin and expanded for 8 days prior to freezing into aliquots of 6 × 10^7^ cells/ml (500× sgRNA representation) for library selection. Before use in library selection, aliquots were thawed and cells were recovered for 24 h. HlyA-containing supernatant (from WAM 1824 cultures) was used to select library populations at a concentration resulting in ∼95% cytotoxicity of wild-type U-937 cells (Fig. S1B). Following selection, viable cells were recovered and expanded under normal cell culture conditions. A total of 3 × 10^7^ cells were frozen for DNA extraction, and an identical repeat selection with HlyA was performed on 6 × 10^7^ cells. Viable cells from twice-selected populations were recovered and expanded under normal growth conditions to 3 × 10^7^ cells for DNA extraction. Three biological replicates of library selections were performed, with two rounds of selection each (Fig. S1A).

### DNA extraction, sequencing, and identification of selection hits.

Genomic DNA from 3 × 10^7^ cells was extracted from two representative vials of the parental library and the once- or twice-selected library populations for each replicate using the Qiagen Midi blood and cell culture kit according to the manufacturer’s protocol. To maintain diversity of the populations, 13 PCRs were performed for the parental populations and three PCRs for selected populations according to the following protocols. Libraries were PCR amplified for 16 cycles with v2adaptor primers (v2adaptor F/R) (see Table S2 in the supplemental material) to generate templates representing sgRNAs present in the different populations. These templates were PCR amplified for 24 cycles with a unique “barcoded” forward primer (F01 to F12) assigned to each library and a universal reverse primer (RUni) (Table S2). PCR products were visualized on a 1.2% agarose–ethidium bromide gel, relative band intensities were quantified by ImageJ, and equimolar amounts from replicate reactions were pooled. Pooled samples were separated on a 1.2% agarose–ethidium bromide gel and extracted with the Qiagen MinElute gel extraction kit, in a final buffer of Tris at pH 8.0 before sequencing. The second set of primers provided adaptors for Illumina sequencing, performed on a HiSeq2500 Rapid at the University of Wisconsin—Madison Biotechnology Core. Sequencing reads were demultiplexed, staggers introduced by unique barcodes were removed, and sequences from individual libraries were downloaded as individual files using the open-source web-based platform Galaxy (usegalaxy.org). Individual library files were used for analysis with MAGeCK as described by the developers (25).

10.1128/mBio.01459-19.6 TABLE S2 Table of primers/oligonucleotides. Download Table S2, PDF file, 0.05 MB. Copyright © 2019 Ristow et al. 2019 Ristow et al. This is an open-access article distributed under the terms of the Creative Commons Attribution 4.0 International license.

### Lentiviral cloning, production, and transduction.

Cloning into lentiCRISPR v2 was performed as described previously (24). Briefly, three sgRNA sequences from the GeCKO v2 library were used per target gene (Table S2). Oligonucleotides (Integrated DNA Technologies) were phosphorylated and annealed before being ligated into purified BsmBI (New England Biolabs)-cleaved and Fast-AP (Fermentas)-dephosphorylated lentiCRISPR v2. Ligation reactions were transformed into chemically competent Mach1 cells (Thermo Fisher Scientific), and plasmid sequences were validated by sequencing. To produce lentivirus, 1 μg each of packaging plasmids psPAX2 and pVSV-G was cotransfected with 1 μg of lentiCRISPR v2 containing sgRNA for target genes into 80% confluent HEK293T cells using TransIT-2020 (Mirus Bio, Madison, WI) according to the manufacturer’s protocol. After 72 h, virus-containing supernatants were harvested, and cell debris was removed by centrifugation at 5,000 × *g* for 5 min. Cleared supernatants were aliquoted and stored at −80°C.

U-937 cells were seeded the day before transduction to obtain 50% confluence the next day. On the day of transduction, 1.5 × 10^6^ cells were pelleted per transduction at 500 × *g* for 5 min. Supernatant was removed, and cells were resuspended directly in 100 μl virus-containing supernatant (described above) with 8 μg/ml Polybrene. Cells were incubated at 37°C for 2 h, gently flicking the tube every 15 min to resuspend cells. After 2 h, cells were transferred to a 12-well plate, and the volume was increased to 2 ml with complete cell medium. Cells recovered under normal growth conditions for 24 h before pelleting cells, discarding supernatant, and resuspending the cells in complete cell medium containing 0.5 μg/ml puromycin. Cells were maintained under normal growth conditions for 2 weeks before limiting dilution into 96-well plates to obtain clonal cell lines. Mutations were assessed by flow cytometry and indel detection by amplicon analysis (IDAA) and confirmed by Sanger sequencing.

Single-gene-knockout U-937 cells were used to generate multisubunit knockouts. Custom gRNA fragments (CRISPR RNA [crRNA; Thermo Fisher]) were complexed with transactivating crRNA (tracrRNA) to generate sgRNAs according to the manufacturer’s protocol. Five micrograms of sgRNA was introduced by nucleofection according to the manufacturer’s protocol (Amaxa nucleofector II; Lonza) into 1 × 10^6^ cells per intended mutation. Cells were recovered in complete medium for 24 h before undergoing limiting dilution into 96-well plates to obtain clonal cell lines. Mutations were assessed by flow cytometry and indel detection by amplicon analysis (IDAA) and confirmed by Sanger sequencing. This process was repeated in a stepwise fashion to obtain triple, quadruple, and quintuple gene knockouts.

### IDAA.

Fifty microliters of cells from growing clonal cell lines was pelleted at 500 × *g* for 10 min in a 96-well PCR plate. Supernatant was removed by flicking the plate, and cells were resuspended in 50 μl QuickExtract DNA extraction solution (Epicentre). DNA was extracted according to the manufacturer’s instructions. To dilute the generally viscous extracts, 150 μl dH_2_O was added to each well, and 1 μl of extracted DNA was used for PCR amplification. Indel detection by amplicon analysis (IDAA) was performed as described previously (27). Briefly, IDAA primers were designed to generate products between 200 and 600 bp surrounding the sgRNA target sequence of interest (Table S2). Forward primers were preceded with an M13 sequence to facilitate a tri-primer reaction to label products for analysis, including target specific forward and reverse primers and a 6-carboxyfluorescein (FAM)-labeled M13 primer in 1:10:10 ratio. PCR was performed in 20-μl reaction mixtures using 2× GoTaq (Promega) using the cycling conditions described in reference 27. Dilutions (1:10) of the PCR product were submitted to the University of Wisconsin—Madison Biotechnology Center for fragment analysis on ABI3730 with a Chimerx Rox625 size standard. Raw data were analyzed with Peak Scanner Software version 3.0 (Applied Biosystems, Life Technologies Corporation).

### Flow cytometry.

Cells were counted on a TC20 cell counter (Bio-Rad), and 1 × 10^6^ cells were stained for analysis. Cells were surface stained with anti-α_L_ (HI111; Biolegend), anti-α_M_ (ICRF44; Biolegend), anti-α_X_ (3.9; Biolegend), and anti-β_2_ (TS1/18, Biolegend; MEM48, Thermo Fisher Scientific) and fixed using IC fixation buffer (Ebioscience). Samples were acquired using an LSRII flow cytometer with at least 50,000 events collected per sample (BD Biosciences) with fluorescence-activated cell sorter (FACS) DIVA software (BD Biosciences). FlowJo software version 10 was used to analyze data (Treestar).

### Far-Western blotting.

Recombinant human integrin α_L_β_2_ (3868-AV), human integrin α_M_β_2_ (4047-AM), human integrin α_X_β_2_ (5755-AX), and human ICAM-1/CD54 (720-IC) were obtained from R&D Systems. Recombinant proteins were resuspended according to the manufacturer’s instructions, aliquoted, and stored at −20°C. Five hundred nanograms of protein was separated per lane of a 4 to 20% gradient SDS-PAGE gel (Bio-Rad). Separated proteins were transferred to nitrocellulose. Membranes were blocked for 1 h, rocking, in 3% bovine serum albumin (BSA)–0.5% Tween 20 in 1× PBS. Membranes were incubated with PEG-precipitated HlyA or LtxA at 1 μg/ml in blocking buffer for 1 h at room temperature, rocking. Membranes were washed 5 times for 1 min each in PBS plus 0.5% Tween 20. Proteins were detected with primary antibodies in blocking buffer for 1 h at room temperature, rocking, against α_L_ (CR38, 1:500; R&D Systems), α_M_ (66519-1, 1:4,000; Proteintech), α_X_ (60258-1, 1:4,000; Proteintech), β_2_ (AF1730, 1:1,000; R&D Systems), anti-HlyA polyclonal antibody (1:5,000 [49]), or a pool of anti-HlyA monoclonal antibodies (B7, 1:10,000; B9, 1:200; B10, 1:200; C7, 1:200, C10, 1:2,000; D1, 1:5,000; E2, 1:800; G3, 1:5,000; G8, 1:2,000; H10, 1:10,000 [49]). Secondary antibodies were diluted in blocking buffer and incubated for 1 h at room temperature, rocking; for the blot against α subunits and HlyA, the antibodies were donkey anti-rabbit 680RD and donkey anti-mouse 800CW, and for the blot against the β_2_ subunit and HlyA or LtxA, the antibodies were donkey anti-goat 800CW and donkey anti-mouse 680RD (all secondary antibodies at 1:10,000; Licor). Anti-HlyA antibodies (monoclonal or polyclonal) equally detected HlyA, whereas only the anti-HlyA monoclonal pool cross-reacted with LtxA, so blots demonstrating direct interaction of the toxins with the β_2_ subunit were performed with the HlyA monoclonal antibody pool and a polyclonal anti-β_2_ subunit antibody. Anti-α subunit antibodies were raised in mice, so the anti-HlyA rabbit polyclonal antibody was used to simultaneously detect both proteins in different channels. Membranes were dried between sheets of Whatman paper, and near-infrared images were captured on a Li-Cor Odyssey Fc imager.

### β_2_ complements.

Full-length β_2_ (amino acids [aa] 1 to 769) or a cytoplasmic tail deletion mutant (β_2_-Δtail, aa 1 to 723) with 7 silent mutations within the 20-bp sgRNA recognition site for sgRNA-ITGB2-1 (to allow complementation without cleavage by the constitutively expressed Cas9 and sgRNA in the parental Δβ_2_ cell line) were synthesized as gBlocks (IDT) with 20-bp overlaps to insert by Gibson assembly (NEB) into a BamHI-digested pcDNA3.1-hygromycin-resistant vector (Thermo Fisher Scientific). DNA was extracted from sequence-confirmed clones and electroporated into a Δβ_2_ (sgRNA-ITGB2-1) cell line with the Mirus Bio Ingenio electroporation kit according to the manufacturer’s instructions using a GenePulser II (Bio-Rad). Complements were selected with 200 μg/ml hygromycin. β_2_ expression was assessed by flow cytometry; bulk populations were sorted for high β_2_ expression, and limiting dilution was performed to obtain single cell clones. Although no clones were obtained that restored wild-type expression of β_2_, the clones with the highest β_2_ expression were assessed for sensitivity to HlyA as described above.
