# Supplementary figures and images for: The Extracellular Domain of the β2 Integrin β Subunit (CD18) Is Sufficient for Escherichia coli Hemolysin and Aggregatibacter actinomycetemcomitans Leukotoxin Cytotoxic Activity
Source: mBio. 2019 Jul 9;10(4):e01459-19. doi: 10.1128/mBio.01459-19 (PMC6747720; doi:10.1128/mBio.01459-19)

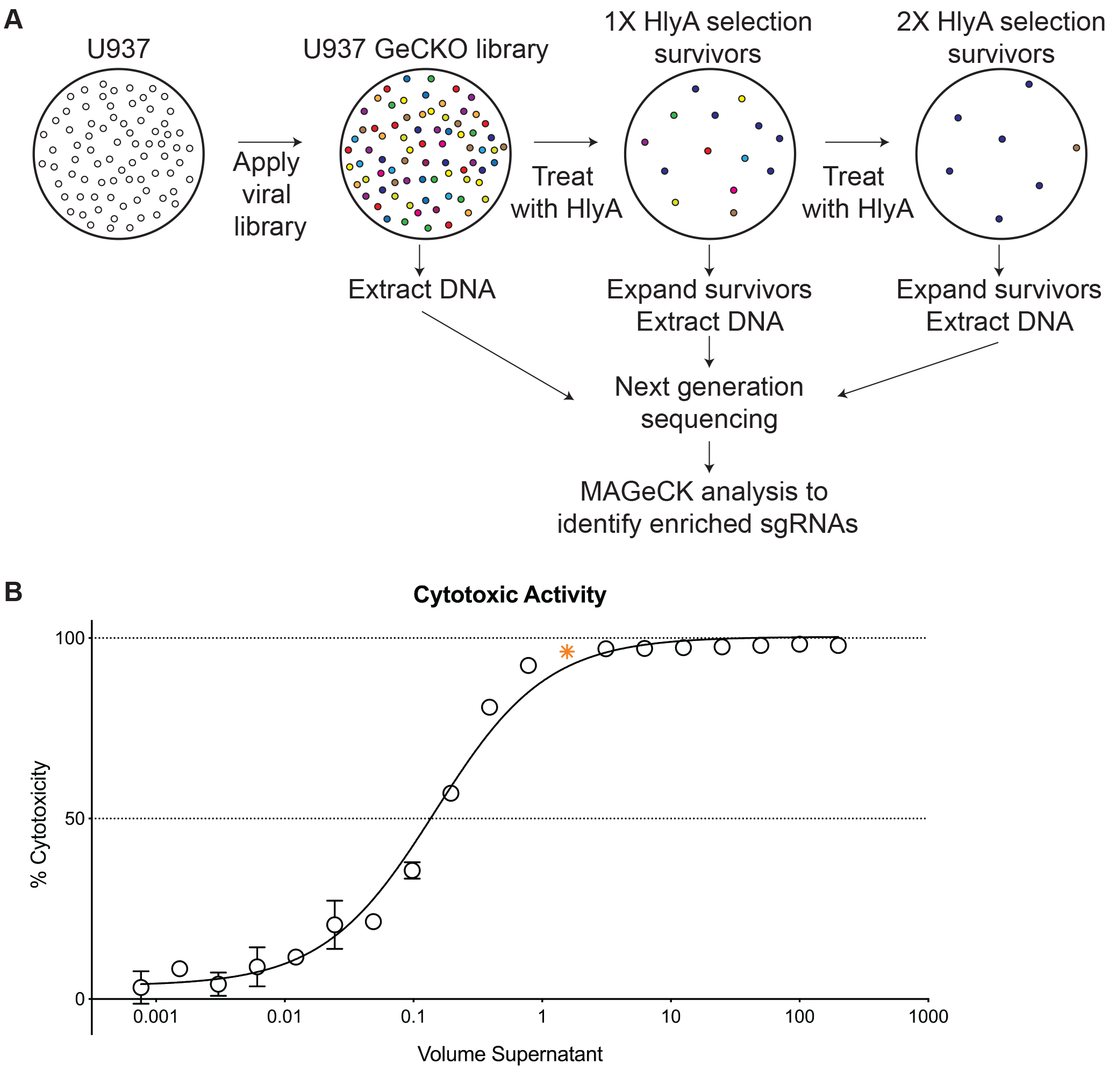

Supplement: FIG S1 [file mBio.01459-19-sf001.tif]

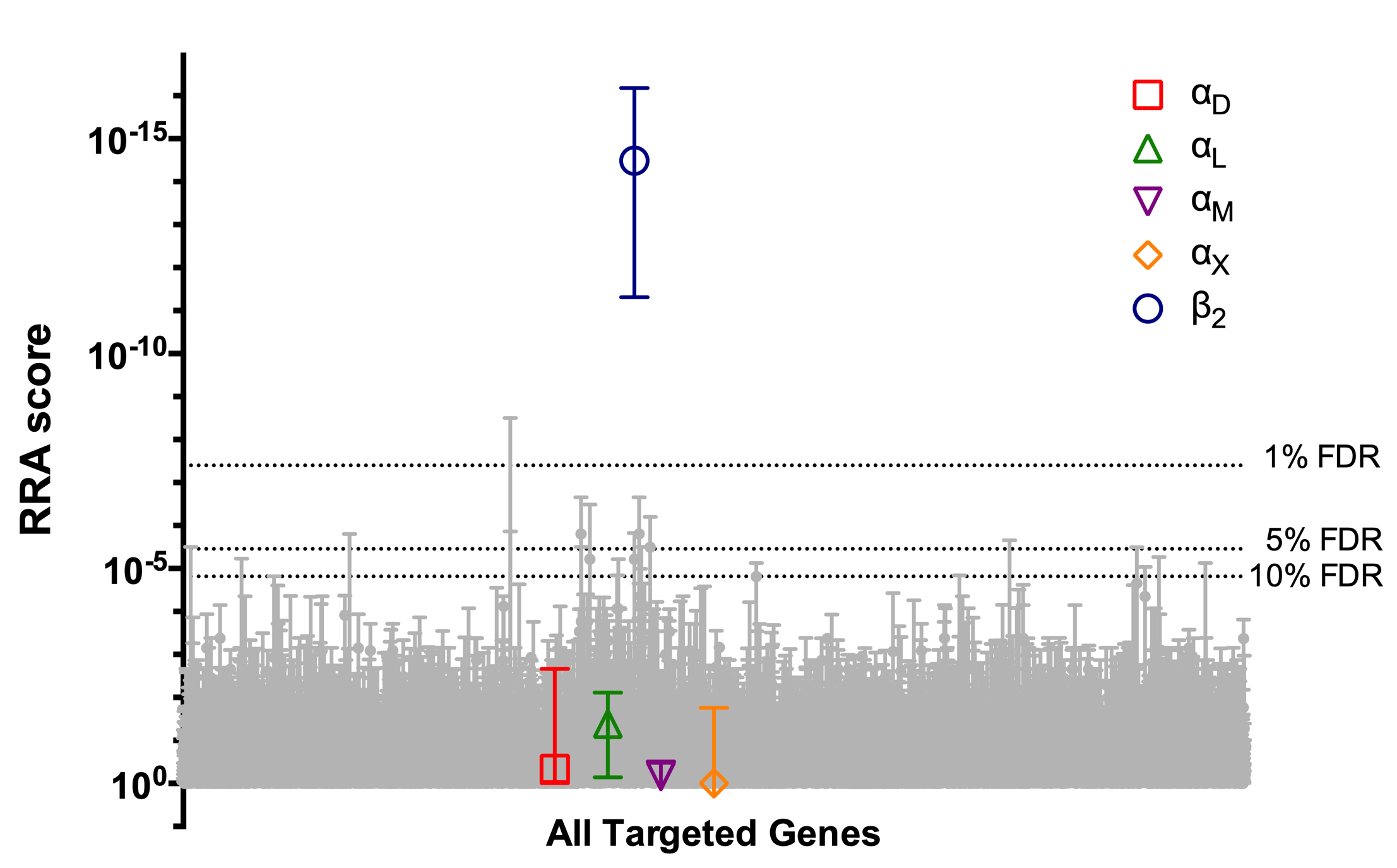

Supplement: FIG S2 [file mBio.01459-19-sf002.tif]

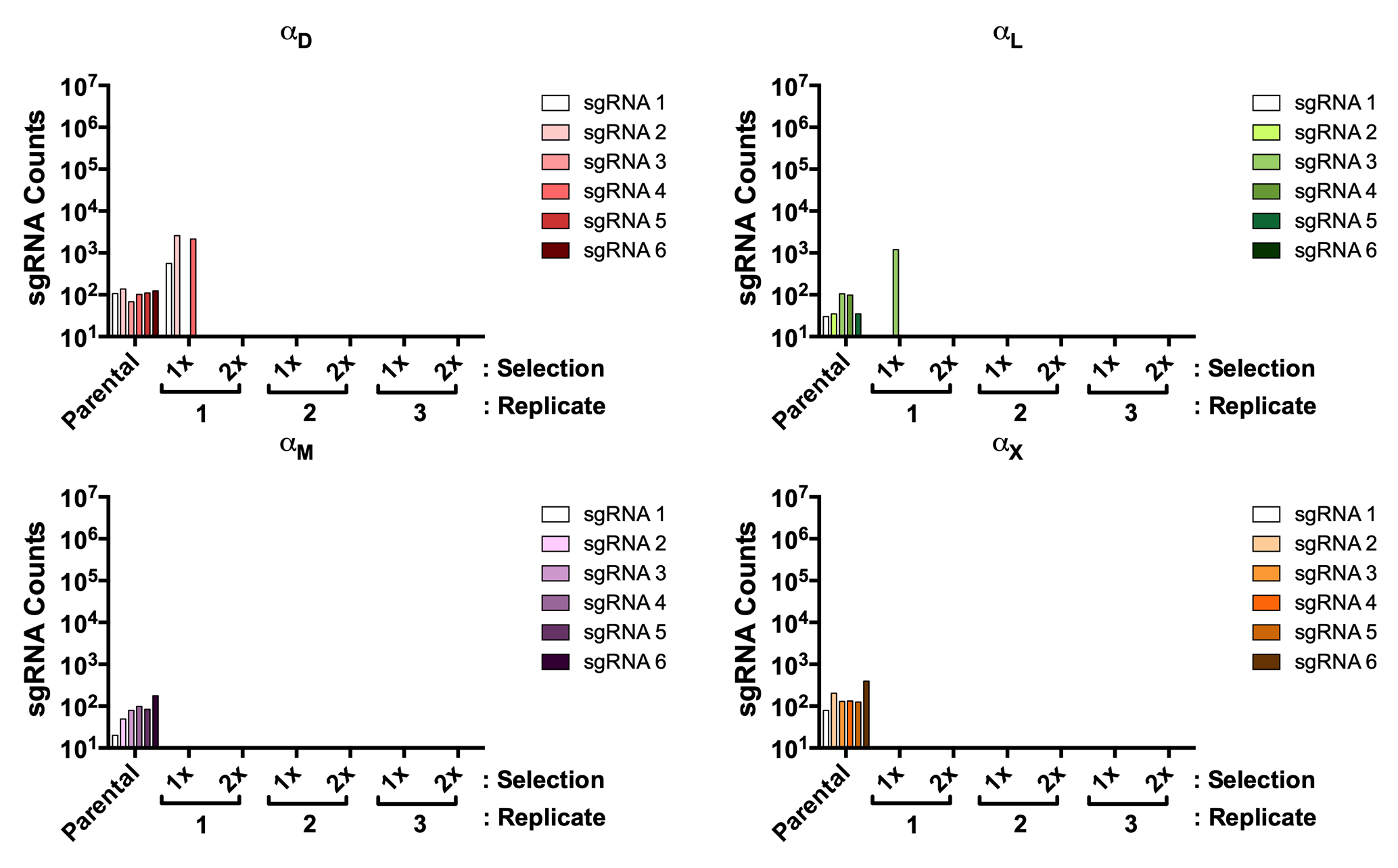

Supplement: FIG S3 [file mBio.01459-19-sf003.tif]

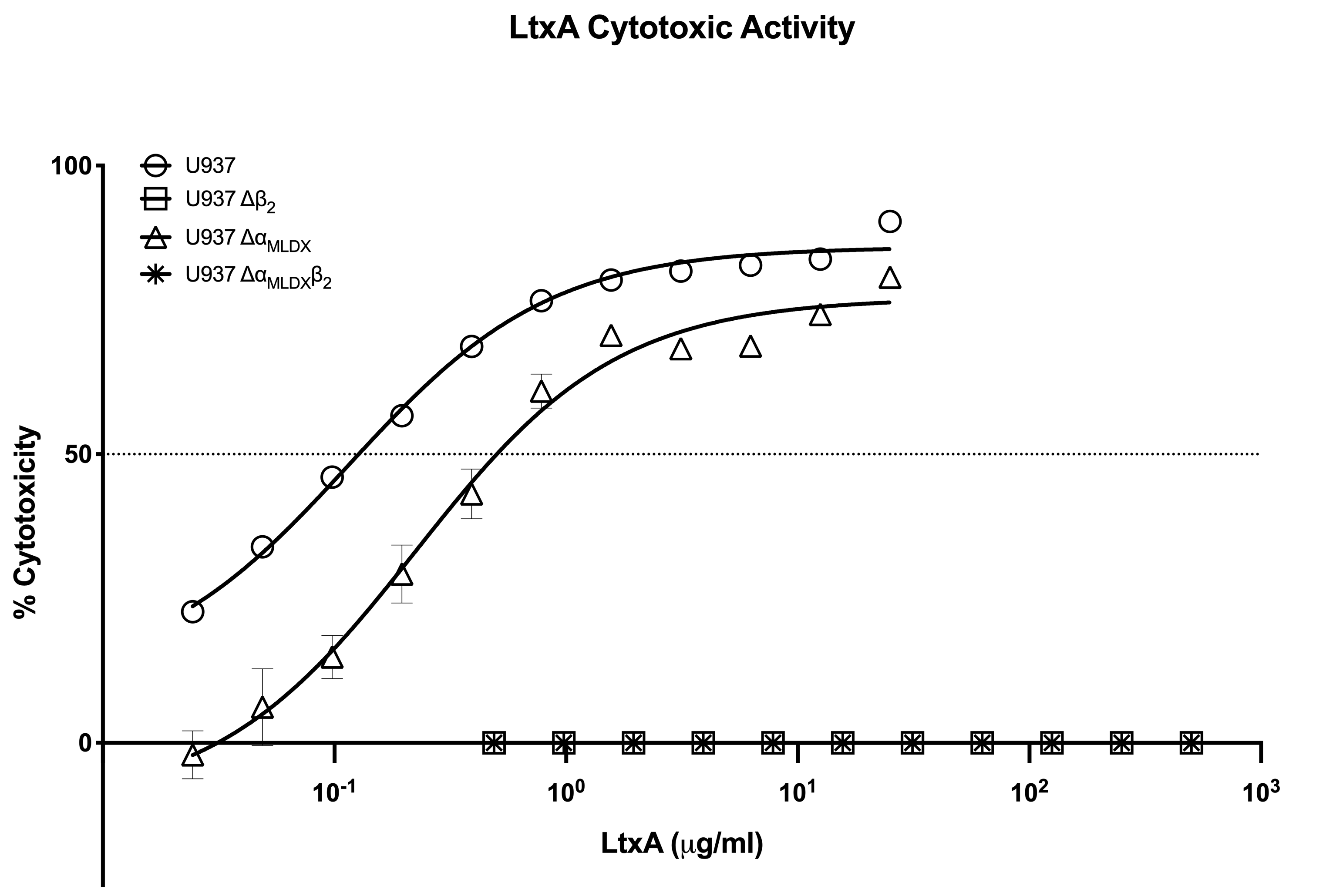

Supplement: FIG S4 [file mBio.01459-19-sf004.tif]
