# Supplementary material for: The Extracellular Domain of the β2 Integrin β Subunit (CD18) Is Sufficient for Escherichia coli Hemolysin and Aggregatibacter actinomycetemcomitans Leukotoxin Cytotoxic Activity
Source: mBio. 2019 Jul 9;10(4):e01459-19. doi: 10.1128/mBio.01459-19 (PMC6747720; doi:10.1128/mBio.01459-19)
Supplement: TABLE S1 [file mBio.01459-19-st001.pdf]

Table S1: Table of mutations in knockout cell lines

Cell name	Target gene	Genetic modification	Sequence
$\Delta\alpha_D$	*ITGAD*	WT sequence	5' GGCCAACCAGACGGGGACGGCTGTATGACT 3'
		-5bp	5' GGCCAACCA - - - - - GGACGGCTGTATGACT 3'
		+1bp	5' GGCCAACCAGACGGGG**G**ACGGCTGTATGACT 3'
$\Delta\alpha_L$	*ITGAL*	WT sequence	5' AGTGTCCTTCTCTCCTGCAAGGTCA 3'
		-2bp	5' AGTGTCCTTCT - - CCTGCAAGGTCA 3'
		-8bp	5' AGTGTCCT - - - - - - - - GCAAGGTCA 3'
$\Delta\alpha_M$	*ITGAM*	WT sequence	5' GTGCGACTACAGCACAGGCTCATGC 3'
		-2bp	5' GTGCGACTACAGCA - - GGCTCATGC 3'
		-1bp	5' GTGCGACTAC - GCACAGGCTCATGC 3'
$\Delta\alpha_X$	*ITGAX*	WT sequence	5' GGAGCTGACAGCCTTCCGTGTGG 3'
		+1bp	5' GGAGCTGACAGCCTTCC**C**GTGTGG 3'
$\Delta\beta_2$	*ITGB2*	WT sequence	5' CAGAACTTCACAGGGCCGGGGGATCCTGACTCCATTCG 3'
		-7bp	5' CAGAACTTCACAGGGCCGGGGGA - - - - - - - - TCCATTCG 3'
		-23bp	5' CAGAACT- TCCATTCG 3'
$\Delta\alpha_{DLMX}$	*ITGAD*	WT sequence	5' TTGTGTCTCCAGACTCGTGGTGGGAGC 3'
		-13bp	5' TTGTGTC - - - - - - - - - - - - - TGGGAGC 3'
		-4bp	5' TTG - - - - TCCAGACTCGTGGTGGGAGC 3'
		+1bp	5' TTGTGTCTCCAG**C**ACTCGTGGTGGGAGC 3'
	ITGAL	WT sequence	5' CAAACTTGAGAGTGTCCTTCTCTCCTGCAAGGTCAG 3'
		-15bp	5' CAACTTG - - - - - - - - - - - - - - - - CCTGCAAGGTCAG 3'
	ITGAM	WT sequence	5' GACTACAGCACAGGCTCATGCGAGCCC 3'
		-3bp	5' GACTACAG - - - AGGCTCATGCGAGCCC 3'
		+3bp	5' GACTACA**CCG**GCACAGGCTCATGCGAGCCC 3'
	ITGAX	WT sequence	5' TGTGGACACGCTGGGTTG 3'
		+1bp	5' TGT**G**GGACACGCTGGGTTG 3'
$\Delta\alpha_{DLMX}\beta_2$ (derived from $\Delta\alpha_{DLMX}$)	*ITGB2*	WT sequence	5' GGGGGATCCTGACTCCATTCGCTGCGACA 3'
		-13bp	5' GGGGGATCC - - - - - - - - - - - - - TGCGACA 3'
		-7bp	5' GGGGGATCC - - - - - - - - ATTCGCTGCGACA 3'
