# Supplementary material for: The Extracellular Domain of the β2 Integrin β Subunit (CD18) Is Sufficient for Escherichia coli Hemolysin and Aggregatibacter actinomycetemcomitans Leukotoxin Cytotoxic Activity
Source: mBio. 2019 Jul 9;10(4):e01459-19. doi: 10.1128/mBio.01459-19 (PMC6747720; doi:10.1128/mBio.01459-19)
Supplement: TABLE S2 [file mBio.01459-19-st002.pdf]

Table S2: Table of primers/oligos

Function	Primer name	Primer sequence (5'-3')
GeCKO library sequencing	V2adaptor_F	AATGGACTATCATATGCTTACCGTAACTTGAAAGTATTTCG
	V2adaptor_R	TCTACTATTCTTTCCCCTGCACTGTtgtgggcgatgtgcgctctg
	F01	AATGATACGGCGACCACCGAGATCTACACTCTTTCCCTACACGACGCTCTTCCGATCTtAAGTAGAGtcttgtggaaaggacgaaacaccg
	F02	AATGATACGGCGACCACCGAGATCTACACTCTTTCCCTACACGACGCTCTTCCGATCTatACACGATCtcttgtggaaaggacgaaacaccg
	F03	AATGATACGGCGACCACCGAGATCTACACTCTTTCCCTACACGACGCTCTTCCGATCTgatCGCGCGGTtcttgtggaaaggacgaaacaccg
	F04	AATGATACGGCGACCACCGAGATCTACACTCTTTCCCTACACGACGCTCTTCCGATCTcgatCATGATCGtcttgtggaaaggacgaaacaccg
	F05	AATGATACGGCGACCACCGAGATCTACACTCTTTCCCTACACGACGCTCTTCCGATCTtcgatCGTTACCAtcttgtggaaaggacgaaacaccg
	F06	AATGATACGGCGACCACCGAGATCTACACTCTTTCCCTACACGACGCTCTTCCGATCTatcgatTCCTTGGTtcttgtggaaaggacgaaacaccg
	F07	AATGATACGGCGACCACCGAGATCTACACTCTTTCCCTACACGACGCTCTTCCGATCTgatcgatAACGCATTtcttgtggaaaggacgaaacaccg
	F08	AATGATACGGCGACCACCGAGATCTACACTCTTTCCCTACACGACGCTCTTCCGATCTcgatcgatACAGGTATtcttgtggaaaggacgaaacaccg
	F09	AATGATACGGCGACCACCGAGATCTACACTCTTTCCCTACACGACGCTCTTCCGATCTacgatcgatAGGTAAGGtcttgtggaaaggacgaaacaccg

	F10	AATGATACGGCGACCACCGAGATCTACACTC TTTCCCTACACGACGCTCTTCCGATCTtAACAA TGGtcttgtggaaaggacgaaacaccg
	F11	AATGATACGGCGACCACCGAGATCTACACTC TTTCCCTACACGACGCTCTTCCGATCTatACTG TATCtcttgtggaaaggacgaaacaccg
	F12	AATGATACGGCGACCACCGAGATCTACACTC TTTCCCTACACGACGCTCTTCCGATCTgatAGG TCGCAtcttgtggaaaggacgaaacaccg
	R universal	CAAGCAGAAGACGGCATACGAGATGTGACTG GAGTTCAGACGTGTGCTCTTCCGATCTTCTAC TATTCTTTCCCCTGCACTGT
sgRNA targets	ITGB2(3) HGLIbA_23779	GCAGCTGATTTCCGGAAACC
	ITGAD(2) HGLibA_23750	AGTCATACAGCCGTCCCGTC
	ITGAD(3) HGLib_23751	TGTGTCTCCAGACTCGTGGT
	ITGAD(4) HGLib_23752	GGACCTACCTGGCGTGGCGT
	ITGAL(1) HGLibB_23723	TACTGACCTTGCAGGAGAGA
	ITGAL(2) HGLibB_23724	GGATTCCTGCATCACTGTGA
	ITGAL(3) HGLibB_23725	TCCCCACAGTTCTTCTCAAA
	ITGAM(2) HGLibA_23761	TACCAGTGCGACTACAGCAC
	ITGAM(3) HGLibB_23726	TGACCTTCCAAGAGAACGCA
	ITGAM(4) HGLibB_23727	CCCATGCACCTGTTAACAGA
	ITGAX(1) HGLibA_23766	GGAGCTGACAGCCTTCCGTG
	ITGAX(2) HGLibB_23732	CAAACCCAGCGCTGTCCACA
	ITGAX(3) HGLibB_23733	GCAGCACTCCTCCTGTTCAC
IDAA primers	ITGAD(1) [sequence ITGAD(2)&(3)] F	GTA AAA CGA CGG CCA GTG AAG TGG CAA ATG CCA GGA ATT TCC C
	ITGAD(1) [sequence ITGAD(2)&(3)] R	AAG CGG GGG CAC AGT TGG G
	ITGAD(2) [sequence ITGAD(4)] F	GTA AAA CGA CGG CCA TTC TGC CTC CAG GCC TGT GG
	ITGAD(2) [sequence ITGAD(4)] R	TTC TCT CCT AGC CAC ACT CGG C

	ITGAL(3) [sequence ITGAL(2)] F	GTA AAA CGA CGG CCA TGT CTA GGT TGC CAG CAA ATC
	ITGAL(3) [sequence ITGAL(2)] R	CCC CAT ATC CCT AAG GCC AAT
	ITGAL(4) [sequence ITGAL(1)&(3)] F	GTA AAA CGA CGG CCA TGG GAG GTA AGA GGG GAG AA
	ITGAL(4) [sequence ITGAL(1)&(3)] R	CCA AGA GAG GGA AGT GGT GT
	ITGAM(3) [sequence ITAM(2)] F	GTA AAA CGA CGG CCA ATC CGG GTA TGG GCC CCC
	ITGAM(3) [sequence ITAM(2)] R	AAG GAA CAC AGA GGG GTT GCC
	ITGAM(4) [sequence ITAM(4)] F	GTA AAA CGA CGG CCA TCT TTG CTT TGG TGG CTT CC
	ITGAM(4) [sequence ITAM(4)] R	TGT GGT CAT TCA TCC AGG CT
	ITGAM(5) [sequence ITAM(3)] F	GTA AAA CGA CGG CCA TGC TTG ATC CTT CCC CCA TT
	ITGAM(5) [sequence ITAM(3)] R	ATT TGT GAG GTC CCT CCT GG
	ITGAX(1) [sequence ITGAX(3)] F	GTA AAA CGA CGG CCA TTC TTC CTT CCC CTG GCC ACC
	ITGAX(1) [sequence ITGAX(3)] R	ATC TCA GAC CCT ATG TGC CTG C
	ITGAX(2) [sequence ITGAX(1)&(2)] F	GTA AAA CGA CGG CCA ATG AGT TGG GTG TCC AGA AGA CC
	ITGAX(2) [sequence ITGAX(1)&(2)] R	TAG CTG TGA GAC CGT GCC TGC
	ITGB2(5) [sequence ITGB2(1)&(2)] F	GTA AAA CGA CGG CCA AGT AGG TCC TGA GTC CTT TCT GC
	ITGB2(5) [sequence ITGB2(1)&(2)] R	TTC TGG GCA GCC CTG ACA CC
	ITGB2(6) [sequence ITGB2(3)] F	GTA AAA CGA CGG CCA CAC CCT GAT AAG CTG CGA AA
	ITGB2(6) [sequence ITGB2(3)] R	TTG GCC GCT CGC AAA ATG TTT AGG
